# Influence of *Lactobacillus plantarum* P-8 on Fermented Milk Flavor and Storage Stability

**DOI:** 10.3389/fmicb.2018.03133

**Published:** 2019-01-09

**Authors:** Tong Dan, Haiyan Chen, Ting Li, Jiale Tian, Weiyi Ren, Heping Zhang, Tiansong Sun

**Affiliations:** Key Laboratory of Dairy Biotechnology and Engineering, Ministry of Education, Key Laboratory of Dairy Products Processing, Ministry of Agriculture, Inner Mongolia Agricultural University, Hohhot, China

**Keywords:** fermented milk, *L. plantarum* P-8, SPME–GC–MS, volatile flavor compounds, storage stability

## Abstract

Previously, we demonstrated that the flavor of milk fermented with *Lactobacillus delbrueckii* subsp. *bulgaricus* (IMAU20401) and *Streptococcus thermophilus* (IMAU40133) at a 1:1000 ratio was superior to that of other ratios of the two strains. In this study, *Lactobacillus plantarum* P-8 was used as the probiotic bacterium. Six ratios (1:1, 1:5, 1:10, 1:50, 1:100, and 1:1000) of *L. plantarum* P-8 to yogurt starter were evaluated. A total of 66 volatile compounds including aldehydes, ketones, acids, alcohols, esters, alcohols, and aromatic compounds were identified in milk fermented with the six different *L. plantarum* P-8 to yogurt starter ratios at 0 d of storage. In particular, key flavor compounds, such as 3-methylbutanal, hexanal, (E)-2-octenal, nonanal, 2-heptanone, 2-nonanone, and acetoin, were identified in the 1:100 ratio treatment. Furthermore, the viable cell count, pH, titratable acidity, viscosity, and syneresis of the milk samples were analyzed during fermentation over 14 d of storage at 4°C. The results indicated that milk can be fermented with *L. plantarum* P-8 in combination with *S. thermophilus* and *L. delbrueckii* subsp. *bulgaricus*, and the physicochemical characteristics of the milk were not affected by the probiotic bacteria.

## Introduction

Probiotics are live microorganisms that confer health benefits to a host when they are consumed in adequate amounts (Food and Agriculture Organization of the United Nations/World Health Organization [FAO/WHO], 2006). Yogurt, which is considered to be a source of probiotics, is made from milk by adding starter cultures and is valued for its unique flavor, desirable texture, and nutritional value (Manilópez et al., 2014). However, there has been some debate regarding the survival of yogurt starter bacteria, including *Streptococcus thermophilus* and *Lactobacillus delbrueckii* subsp. *bulgaricus*, which have the ability to survive gastric passage to colonize the gut (Mater et al., 2010). Probiotic bacteria are mostly consumed as a component of food and must overcome physical and chemical barriers in the gastrointestinal tract, particularly acid and bile stresses (Tamang et al., 2016a). Today, it is common to find yogurt and fermented milk products that contain probiotic bacteria in the market, such as Jelley Brown (United States) and Zott (Germany), which have added *Lactobacillus acidophilus*, or Yili Changqing (China), which has added *Bifidobacterium* and *Lactobacillus rhamnosus.*

Research over the past decade has demonstrated the health benefits of probiotic bacteria such as *Lactobacillus* and *Bifidobacterium* (Bao et al., 2010; Ashraf and Shah, 2011), including antioxidant properties (Zhang et al., 2017) and effects on lowering blood pressure (He et al., 2017), reducing serum cholesterol levels (Guan et al., 2017), and stimulating the immune system (Ashraf and Shah, 2014). *L. plantarum* is distributed worldwide and is present in meat, fish, dairy products, and plant-based fermented foods (Siezen et al., 2010; Tamang et al., 2016b; Shangpliang et al., 2018). *Lactobacillus plantarum* P-8 was isolated from traditional fermented milk. It possesses excellent fermentation properties and is considered to be a probiotic bacterium (Bao et al., 2012a,b; Zhang et al., 2015). The complete genome of *L. plantarum* P-8 consists of a circular 3.03 Mb chromosome and seven plasmids (Bao et al., 2012a). *L. plantarum* P-8 can significantly reduce lipid levels, enhance immune function, and improve the intestinal microbiome (Bao et al., 2012b; Zhang et al., 2015). In addition, *L. plantarum* P-8 can be used synergistically with *S. thermophilus* as a starter to improve the flavor and texture of fermented dairy products (He et al., 2012). However, the relationship between fermented milk quality and probiotic effects is poorly understood.

Solid-phase microextraction coupled with gas chromatography–mass spectrometry (SPME–GC–MS) has been used extensively to analyze flavor compounds, including those in fermented milk (Pan et al., 2014), goat milk cheese (Chiofalo et al., 2004), and fermented soymilk (Yin et al., 2013). The combined fermentation of probiotics and yogurt starters can improve the health benefits and flavor profile of fermented milk. Due to its probiotic properties, *L. plantarum* P-8 has been used extensively in the production of dairy products such as fermented soymilk (Wang et al., 2013) and fermented milk (Guo et al., 2013). As living standards improve, consumers place greater value on the flavor and probiotic content of fermented milk when choosing such drinks. The objective of this study was to evaluate the flavor and shelf life, as well as the pH, titratable acidity (TA), viable cell counts, viscosity, and syneresis, of milk fermented using a 1:100 ratio of *L. plantarum* P-8 to *S. thermophilus* and a 1:1000 fixed ratio of *L. delbrueckii* subsp. *bulgaricus* to *S. thermophilus* during 14 d of storage at 4°C.

## Materials and Methods

### Strain Culture and Reagents

*Streptococcus thermophilus* (IMAU40133), *L. delbrueckii* subsp. *bulgaricus* (IMAU20401), and *L. plantarum* P-8 were obtained and cryopreserved from the Lactic Acid Bacteria Collection Center of Inner Mongolia Agricultural University. These isolates were activated in M17 (HB0391, QuingDoa HopeBiol Co., Quingdau, China) and De Man, Rogosa, and Sharpe (MRS) (027312, Huankai Microbial, Guangdong, China) liquid media at 37°C for 24 h, respectively. After subculturing in 50 ml M17 and 500 ml MRS media for two consecutive passages at 37°C for 24 h, the cells were collected and resuspended in PBS buffer (0.8% NaCl, 0.02% KH_2_PO_4_, 0.115% Na_2_HPO_4_, 1% tryptone, and 0.1% sodium glutamate inactivated at 121°C for 15 min). 1,2-Dichloro-benzene, which was used as an internal standard (ISTD), was purchased from Sigma-Aldrich (Steinheim, Germany). MRS broth and whole milk powder were purchased from OXOID (Hampshire, United Kingdom) and NZMP (Wellington, New Zealand), respectively.

### Fermented Milk Manufacture

Whole milk powder (11.5%) was stirred and dissolved in distilled water at 50°C. The water temperature was increased to 60°C, and 6.5% sucrose was added and mixed well and then hydrated for 30 min. Homogenization was performed twice in succession (65°C at 15 and 35 MPa, respectively) by high-pressure homogenization (Shanghai, China), and the resulting homogenized milk was pasteurized at 95°C for 5 min and quickly cooled in ice water to 4°C until use. The yogurt starters were compounded from *L. delbrueckii* subsp. *bulgaricus* (IMAU20401) isolated from traditional fermented dairy products and *S. thermophilus* 40133 at a 1:1000 ratio (Dan et al., 2017b). *L. plantarum* P-8 cultures were compounded with the yogurt starters at ratios of 1:1, 1:5, 1:10, 1:50, 1:100, and 1:1000. Using the amount of *S. thermophilus* (40133) added to reach 5 × 10^7^ CFU/ml as the benchmark, *L. delbrueckii* subsp. *bulgaricus* (IMAU20401) and *L. plantarum* P-8 were added to the homogenized whole milk, which was added to a 15 ml gas-phase flask and fermented in an incubator at 42°C. When the pH of the sample reached 4.5 and the TA reached 70–90°C, the milk was transferred to 4°C for storage (0 d) to determine the volatile flavor compounds.

### Physicochemical Characteristics of Fermented Milk

#### Determination of pH

The pH of the fermented milk was measured at 20°C using a pHSJ-3F pH meter (Leici, Shanghai, China) in parallel.

#### Determination of TA

A 5 g sample of the fermented milk was weighed accurately using an electronic balance and placed in a 100 ml conical flask. To the conical flask, 20 ml CO_2_-free distilled water and three drops of phenolphthalein indicator agent were added, and the flask was shaken well. A 0.1 mol/l NaOH standard solution was added for titration until a reddish color developed. If the color of the solution did not disappear within 30 s, the volume of the NaOH standard solution added was recorded. Triplicates of each fermented milk sample were performed in parallel, and the following formula was used:

$$\begin{array}{c} X=\frac{c\times V\times 100}{m\times 0.1}, \end{array}$$

where “*X*” represents the acidity of the fermented milk sample in degrees (°T), “*c*” represents the molar concentration (mol/l) of the NaOH standard solution, “*V*” represents the volume (ml) of the NaOH standard solution consumed at time of titration, “*m*” represents the mass (g) of the sample, and 0.1 is the molar concentration (mol/l) of NaOH, as defined by the acidity theory.

#### Determination of Viable Cell Counts

The fermented milk sample (0.5 ml) was placed in 4.5 ml of sterilized physiological saline and the mixture was shaken to mix well. A serial dilution was performed. Viable bacterial counts of *S. thermophilus* 40133, *L. delbrueckii* subsp. *bulgaricus* IMAU20401, and *L. plantarum* P-8 in the fermented milk were determined by culturing the diluted samples at 37°C in an incubator for 48 h using the MRS solid medium decanter method and counting the resulting colonies.

#### Determination of Viscosity

The fermented milk (40 ml) was centrifuged in triplicate using a viscometer at 20–22°C at 100 rpm for 30 s.

#### Determination of Syneresis

A 20 g sample of fermented milk was weighed and placed in a funnel with a piece of filter paper (New Star Medium-Speed Qualitative Filter Paper, Hangzhou Special Paper Industry, Hangzhou, China) and allowed to stand at 4°C for 2 h. The filtrate was collected and weighed. The following formula was used to calculate syneresis:

$$\begin{array}{c} \mathrm{Syneresis}(\%)\;=\;\mathrm{Filtrateweight}(g)/Sampleweight(g)\;\times \;100\%. \end{array}$$

#### Determination of Volatile Flavor Compounds

##### Isolation of volatile flavor compounds

The SPME fibers were inserted into the injection port of the Agilent 7890B gas chromatograph (Agilent Technologies Inc., Palo Alto, CA, United States) at 250°C for 5 min for preconditioning. They were then inserted above the gas-phase bottle for extraction for 60 min. Desorption was conducted at 250°C for 3 min.

A temperature-programmed route was used for chromatography. The temperature was maintained at 35°C for 3 min and then increased by 4°C/min to 140°C. The temperature was maintained at 140°C for 1 min and increased to 250°C for 3 min. The transfer line temperature was set to 250°C. The carrier gas was helium, the flow rate was 1.0 ml/min, and no split sampling was performed.

For MS, electron ionization was performed at 70 eV. The ion source temperature was 230°C, the mass scan range was *m*/*z* 33–450 AMU, and the emission current was 100 μA.

#### Qualitative Analysis

We used the National Institute of Standards Technology Mass Spectral Database 11 to reference the published literature and identify compounds. We calculated the relative peak area ratio of all components based on normalization of the peak area (the percentage of each component’s peak area relative to the total peak areas for all substances in the ion chromatograms). We calculated the retention index of each component using a temperature-programmed method to identify the compounds. The retention index (RI) was determined by the following equation:

$$\begin{array}{c} \mathrm{RI}=100\times \left[z+\frac{{\mathrm{RT}}_{\left(X\right)}-{\mathrm{RT}}_{\left(Z\right)}}{{\mathrm{RT}}_{\left(Z+1\right)}-{\mathrm{RT}}_{\left(Z\right)}}\right], \end{array}$$

where “RT” represents the retention time (min) and the retention times according to the carbon number of *n*-alkanes follow the order RT (*z*) < RT (*X*) < RT (*Z* + 1). *n*-Alkane standards (C3–C25) were obtained from AccuStandard (New Haven, CT, United States).

1,2-Dichlorobenzene solution (Sigma-Aldrich, St. Louis, MO, United States) was added to the fermentation sample as the ISTD. The concentrations of all flavor components in the samples were used in the following formula to calculate the concentration of each compound:

$$\begin{array}{c} c_{i}=\frac{A_{i}}{A_{s}}\times c_{s}, \end{array}$$

where “*c_i_*” represents the concentration (μg/l) of the compound in the test sample, “*c_s_*” represents the concentration (μg/l) of 1,2-dichlorobenzene, “*A_i_*” represents the chromatographic peak area of the test substances in the sample, and “*A_s_*” represents the chromatographic peak area of the ISTD.

#### Evaluation of Odor Activity

To quantify the volatile flavor compounds in the fermented milk, we used the flavor threshold value for each flavor compound in water and calculated the physical parameters of the compounds, namely the odor activity value (OAV), which indicates the flavor contribution from each flavor compound. The following formula was used:

$$\begin{array}{c} {\mathrm{OAV}}_{i}=\frac{C_{i}}{{\mathrm{OT}}_{i}}, \end{array}$$

where OAV*_i_* represents the flavor of compound *i, C_i_* represents the concentration of compound *i* in fermented milk (μg/l), and OT*_i_* represents the flavor threshold value of the compound in water.

### Sensory Evaluation

A total of 10 trained panelists conducted a sensory assessment of the flavor of the milk samples fermented with different *L. plantarum* P-8 to yogurt starter ratios at 0 d of storage, based on the requirements specified by RHB 103-2004 of China’s dairy industry for assessing the sensory quality of cultured milk.

### Statistical Analysis

Data were analyzed using Microsoft Excel, SPSS v19.0, SIMCA-P v11.5, and SAS v9.0. Normalized data were assessed by principal component analysis, significance tests, and correlation analysis. Principal component analysis was performed to determine the most important volatile compounds in milk fermented with the six different ratios of *L. plantarum* P-8 to yogurt starter. We used Origin v8.6 and Heml v1.0 to create principal component loading plots and score plots. Similarities were analyzed in the chromatograms obtained from the fermented milk samples using the Similarity Evaluation System for Chromatographic Fingerprint of Traditional Chinese Medicine (version A) and GC fingerprints were obtained.

## Results

### Volatile Flavor Compounds in Fermented Milk

*Lactobacillus plantarum* P-8 was compounded and fermented using *L. delbrueckii* subsp. *bulgaricus* and *S. thermophilus* (1:1000) yogurt starter at six different inoculation ratios (1:1, 1:5, 1:10, 1:50, 1:100, and 1:1000). At 0 d of storage, 66 volatile flavor compounds were identified in milk fermented with the six different ratios of probiotic bacteria using the HS–SPME–GC–MS technique (Table 1). These compounds included various types of aldehydes, ketones, carboxylic acids, alcohols, esters, and aromatic hydrocarbons.

**Table 1:** Volatile compounds produced by milk fermented with different ratios of *L. plantarum* P-8 to starter culture at 0 d of storage.

No.	Volatile compound	Chemical formula	RT^1^	RI^2^	RI^3^	Method^4^	μg/l					
							1:1	1:5	1:10	1:50	1:100	1:1000
**Aldehyde compounds**												
1	3-Methyl-butanal	C_5_H_10_O	3.63	700.59	697	MS, RI	–	2.28 ± 0.002	2.02 ± 0.001	5.18 ± 0.034	8.51 ± 0.006	11.32 ± 0.053
2	Hexanal	C_6_H_12_O	6.86	809.1	809	MS, RI	–	–	5.5 ± 0.09	15.3 ± 0.517	32.96 ± 0.067	32.08 ± 0.085
3	(E)-2-Hexenal	C_6_H_10_O	8.97	864.56	861	MS, RI	1.17 ± 0.103	0.65 ± 0.174	0.67 ± 0.062	1.47 ± 0.258	2.9 ± 0.713	2.73 ± 0.507
4	(Z)-4-Heptenal	C_7_H_12_O	10.39	901.97	902	MS, RI	–	–	–	0.44 ± 3.462	–	0.75 ± 0.287
5	Heptanal	C_7_H_14_O	10.85	914.27	910	MS, RI	1.51 ± 0.103	2.79 ± 0.000	2.89 ± 0.004	3.76 ± 0.705	8.72 ± 0.902	8.42 ± 0.318
6	(Z)-2-Heptenal	C_7_H_12_O	12.87	968.82	–	MS	5.61 ± 0.051	–	–	1.06 ± 0.603	–	7.82 ± 0.804
7	(E)-2-Heptenal	C_7_H_12_O	12.87	968.86	967	MS, RI	–	–	3.44 ± 0.001	–	10.3 ± 0.519	9.24 ± 0.702
8	(E,E)-2,4-Heptadienal	C_7_H_10_O	14.81	1023.72	1023	MS, RI	–	2.16 ± 1.068	0.83 ± 0.309	2.67 ± 0.405	3.76 ± 2.001	4.86 ± 0.004
9	Benzaldehyde	C_7_H_6_O	12.94	970.7	970	MS, RI	0.46 ± 0.079	0.74 ± 0.007	0.81 ± 0.043	1.08 ± 0.025	0.65 ± 0.002	–
10	(E)-2-Octenal	C_8_H_14_O	16.4	1071.64	1065	MS, RI	5.51 ± 0.06	3.81 ± 0.051	3.8 ± 0.051	4.96 ± 0.3615	10.03 ± 0.405	8.03 ± 1.280
11	Nonanal	C_9_H_18_O	17.92	1118.74	1119	MS, RI	1.03 ± 0.071	1.05 ± 0.069	0.63 ± 0.194	0.56 ± 0.003	2.38 ± 0.051	1.99 ± 0.147
12	(E)-2-Nonenal	C_9_H_16_O	19.65	1175.39	1174	MS, RI	–	3.67 ± 0.372	–	–	–	–
13	(E)-2-Decenal	C_10_H_18_O	22.51	1274.41	1279	MS, RI	–	7.69 ± 2.826	–	–	–	–
14	(Z)-2-Decenal	C_10_H_18_O	22.61	1277.94	1280	MS, RI	12.53 ± 0.921	9.89 ± 0.724	9.58 ± 0.003	9.38 ± 0.035	19.7 ± 0.084	15.6 ± 6.932
15	2-Undecenal	C_11_H_20_O	24.72	1355.87	1359	MS, RI	–	0.69 ± 0.047	1.04 ± 0.078	–	–	–
16	(E)-2-Undecenal	C_11_H_20_O	25.29	1377.31	1374	MS, RI	1.66 ± 0.229	1.00 ± 0.173	–	1.50 ± 0.145	–	1.46 ± 0.042
17	(E)-2-Dodecenal	C_7_H_14_O	27.43	1452.66	1452	MS, RI	–	–	–	14.6 ± 0.029	2.57 ± 0.073	1.76 ± 0.132
**Ketone compounds**												
18	3-Methyl-2-butanone	C_5_H_10_O	3.19	667.69	666.1	MS, RI	–	–	–	0.46 ± 0.025	0.77 ± 0.115	0.89 ± 0.023
19	Acetoin	C_4_H_8_O_2_	4.08	716.09	712	MS, RI	–	–	–	–	15.5 ± 0.097	–
20	2-Heptanone	C_7_H_14_O	10.42	902.86	902	MS, RI	13.55 ± 0.270	10.54 ± 0.034	8.16 ± 0.027	3.03 ± 0.158	27.84 ± 0.395	25.18 ± 0.906
21	5-Methyl-3-heptanone	C_8_H_16_O	12.71	964.55	962	MS, RI	0.5.0 ± 0.851	–	0.46 ± 0.004	11.11 ± 0.016	0.82 ± 0.072	–
22	2-Propyl-1-heptanone	C_10_H_22_O	12.94	970.85	–	MS, RI	2.07 ± 0.048	1.38 ± 0.009	1.33 ± 0.026	0.92 ± 0.007	3.78 ± 0.003	4.52 ± 0.165
23	2-Nonanone	C_9_H_18_O	17.53	1106.15	1104	MS, RI	13.27 ± 0.004	9.94 ± 0.058	8.44 ± 0.029	1.77 ± 0.076	21.7 ± 0.148	18.17 ± 0.009
24	2-Undecanone	C_11_H_22_O	23.52	1310.53	1305	MS, RI	2.31 ± 0.047	1.65 ± 0.064	1.79 ± 0.005	1.99 ± 0.092	3.45 ± 0.036	2.76 ± 0.005
**Carboxylic acids**												
25	3-Heptenoic acid	C_7_H_12_O_2_	11.82	940.54	947	MS, RI	–	–	–	0.47 ± 0.085	0.78 ± 0.172	–
26	Hexanoic acid	C_6_H_12_O_2_	14.37	1010.42	1013	MS, RI	6.88 ± 0.427	1.45 ± 0.005	4.24 ± 0.044	4.29 ± 0.018	–	–
27	Heptanoic acid	C_7_H_14_O_2_	16.24	1063.81	1065	MS, RI	–	–	–	2.86 ± 0.138	5.64 ± 0.004	5.16 ± 0.032
28	7-Oxo-octanoic acid	C_8_H_14_O_3_	17.4	1101.69	–	MS, RI	1.15 ± 0.067	1.19 ± 0.205	–	–	2.7 ± 0.105	3.95 ± 1.312
29	Cyclohexanecarboxylic acid	C_7_H_12_O_2_	19.17	1159.69	1157	MS, RI	–	–	0.27 ± 0.065	–	–	–
30	2-Undecenoic acid	C_11_H_20_O_2_	22.59	1277.25	–	MS	1.58 ± 0.015	–	1.11 ± 0.007	0.79 ± 0.320	1.46 ± 0.405	0.84 ± 0.018
31	Z-8-Methyl-9-tetradecenoic acid	C_15_H_28_O_2_	33.17	1727.78	–	MS	1.68 ± 0.402	–	9.06 ± 5.004	2.79 ± 0.108	–	–
**Alcohol compounds**											
32	3-Methyl-1-butanol	C_5_H_12_O	5.2	752	749	MS, RI	2.67 ± 0.436	1.87 ± 0.270	0.86 ± 0.089	–	4.25 ± 0.371	4.43 ± 0.001
33	Dicyclopropyl carbinol	C_7_H_12_O	6.53	800.26	–	MS	0.43 ± 0.054	–	–	–	–	1.01 ± 0.054
34	4-Hepten-1-ol	C_7_H_14_O	9.26	872.19	870	MS, RI	0.81 ± 0.006	–	–	34.73 ± 0.104	1.53 ± 0.241	1.61 ± 0.003
35	Hexanol	C_6_H_14_O	9.65	882.36	880	MS, RI	15.6 ± 0.190	11.71 ± 0.165	8.53 ± 0.054	5.4 ± 0.208	23.84 ± 0.002	22.33 ± 0.418
36	2-Ethenyl-bicyclo [2.1.1]hexan-2-ol	C_6_H_14_O	9.65	882.5	880	MS, RI	–	–	–	13.01 ± 0.154	1.08 ± 0.208	0.63 ± 0.002
37	(Z)-3-Hepten-1-ol	C_7_H_14_O	12.36	955.11	959	MS, RI	–	0.32 ± 0.418	–	–	–	–
38	*cis*-Hept-4-enol	C_7_H_14_O	13.26	979.26	–	MS	0.99 ± 0.454	0.62 ± 2.343	–	0.87 ± 0.007	–	1.39 ± 0.903
39	Heptanol	C_7_H_16_O	13.43	983.99	975	MS, RI	33.63 ± 0.438	27.2 ± 0.117	22.75 ± 0.004	1.43 ± 0.205	64.93 ± 0.437	59.65 ± 0.005
40	1-Octen-3-ol	C_8_H_16_O	13.75	992.48	991	MS, RI	1.34 ± 0.101	1.14 ± 0.392	0.77 ± 0.052	0.54 ± 0.006	2.28 ± 0.181	2.63 ± 0.060
41	3-Methyl-hepta-1,6-dien-3-ol	C_8_H_14_O	14.01	999.53	–	MS	1.3 ± 0.001	–	0.82 ± 0.114	–	–	1.89 ± 0.120
42	3-Decyn-2-ol	C_10_H_18_O	14.26	1007.1	1101	MS, RI	–	0.51 ± 0.004	–	–	–	–
43	3,5-Octadien-2-ol	C_8_H_14_O	15.41	1041.72	1039	MS, RI	0.56 ± 0.187	0.46 ± 0.405	–	0.78 ± 0.009	1.07 ± 0.203	1.06 ± 0.158
44	(Z)-2-Octen-1-ol	C_8_H_16_O	16.07	1061.59	1067	MS, RI	0.55 ± 0.903	–	–	–	0.71 ± 0.002	0.68 ± 0.194
45	9-Oxabicyclo[6.1.0] nonan-4-ol	C_8_H_14_O_2_	17.69	1111.4	–	MS	1.03 ± 0.146	–	0.38 ± 0.055	–	1.71 ± 0.103	0.84 ± 0.166
46	3,4-Dimethylcyclo hexanol	C_8_H_16_O	18.02	1121.96	–	MS, RI	0.7 ± 0.052	0.3 ± 0.008	0.39 ± 0.049	4.83 ± 0.481	0.96 ± 0.007	0.9 ± 0.173
47	2-Nonen-1-ol	C_9_H_18_O	18.49	1137.37	–	MS, RI	0.55 ± 0.061	–	1.39 ± 1.294	0.48 ± 0.141	–	–
48	(E)-2-Nonen-1-ol	C_9_H_18_O	19.52	1171.14	1171	MS, RI	–	1.33 ± 0.264	–	–	–	–
49	Nonanol	C_9_H_20_O	19.99	1186.62	1186	MS, RI	6.22 ± 0.076	4.74 ± 0.367	3.84 ± 0.043	0.72 ± 0.324	9.74 ± 0.286	8.34 ± 0.006
50	2-Butyl-1-octanol	C_12_H_26_O	25.05	1368.32	–	MS, RI	–	0.97 ± 0.307	3.01 ± 0.256	0.65 ± 0.156	1.7 ± 1.009	–
51	2-Methyl-1-hexadecanol	C_17_H_36_O	34.45	1823.35	–	MS	2.13 ± 0.043	–	3.77 ± 0.627	18.86 ± 0.516	–	–
**Ester compounds**											
52	Butanoic acid, 2-ethyl-1,2,3-propanetriyl ester	C_21_H_38_O_6_	21.46	1237.22	–	MS, RI	1.07 ± 0.003	–	0.74 ± 0.109	0.93 ± 0.007	–	1.18 ± 0.038
53	Allyl 2-ethyl butyrate	C_9_H_16_O_2_	21.86	1251.46	1254	MS, RI	1.03 ± 0.614	–	0.49 ± 0.325	1.35 ± 1.086	1.59 ± 0.156	–
54	Acetic acid, 3,7,11,15-tetramethyl-hexadecyl ester	C_22_H_44_O_2_	34.31	1811.51	–	MS	0.4 ± 0.130	0.45 ± 0.031	0.27 ± 0.298	–	–	–
**Aromatic hydrocarbons**											
55	*n*-Hexane	C_6_H_14_	2.27	–	–	MS	1.89 ± 0.927	0.77 ± 0.316	0.27 ± 0.141	1.68 ± 0.041	–	2.79 ± 0.782
56	Heptane	C_7_H_16_	3.65	701.31	–	MS	1.34 ± 1.483	–	–	–	–	–
57	2,4-Dimethyl-hexane	C_8_H_18_	4.85	742.61	738.9	MS, RI	–	5.22 ± 0.052	–	–	–	–
58	Octane	C_8_H_18_	5.45	763.28	760	MS, RI	3.66 ± 0.003	–	–	–	–	–
59	Octene	C_8_H_16_	6.52	800.18	799	MS, RI	0.62 ± 0.014	–	–	0.42 ± 0.005	0.87 ± 0.018	–
60	1-Nonene	C_9_H_18_	10.15	895.61	893	MS, RI	–	–	–	27.65 ± 0.074	–	–
61	1,2-Dimethyl-cyclooctene	C_10_H_18_	18.53	1138.74	–	MS	–	–	0.2 ± 0.092	–	–	0.97 ± 0.327
62	7-Methyl-3-octyne	C_9_H_16_	18.53	1138.78	–	MS	0.61 ± 0.271	0.55 ± 0.764	–	0.31 ± 0.373	1.08 ± 0.158	–
63	Tetradecane	C_14_H_30_	26.33	1415.01	–	MS, RI	–	0.57 ± 0.089	0.4 ± 0.274	0.65 ± 0.148	0.98 ± 0.231	0.81 ± 0.520
64	Pentadecane	C_15_H_32_	29.27	1520.11	–	MS	–	–	–	1.29 ± 0.589	–	–
65	2,6,10-Trimethyl-tetradecane	C_17_H_36_	31.43	1620.08	–	MS, RI	–	–	–	4.41 ± 0.625	–	–
66	Hexadecane	C_16_H_34_	31.44	1620.2	–	MS	–	0.56 ± 0.259	–	1.65 ± 0.610	–	–

*^*1*^Retention time. ^*2*^Retention indices (RI) of unknown compounds on an HP-5MS column calculated against the GC–MS retention time of *n*-alkanes (C3–C25). ^*3*^RI from database (http://webbook.nist.gov/chemistry). ^*4*^RI, agreed with retention index from the literature; MS, compared with Nist 11 Mass Spectral Database; STD, agreed with the mass spectrum of standard chemical. “–”, not detected.*

### Principal Component Analysis of Volatile Compounds

**FIGURE 1:**
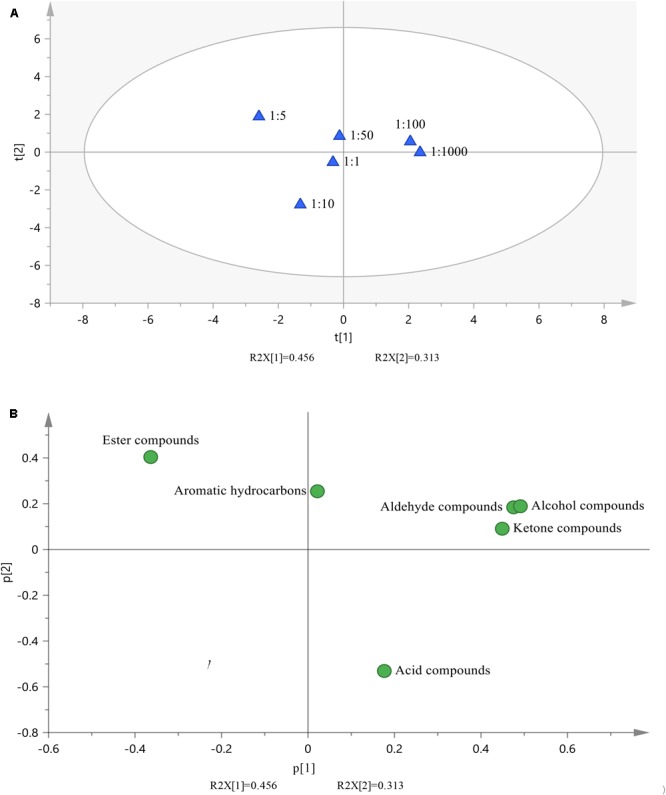
Principal component analysis. **(A)** Scatter plot of the component scores for milk fermented with six different ratios of probiotic strains. **(B)** Scatter plot of the loadings for six classes of volatile compounds.

Principal component analysis was performed to examine the differences among the volatile compounds from milk fermented with different ratios of *L. plantarum* P-8 to starter culture at 0 d of storage. The distribution of the scores in the first two scatter plots (Figure 1A) revealed two separate clusters that corresponded to the six different ratios of the probiotic bacteria. The volatile flavor compounds in milk fermented with the 1:100 and 1:1000 ratios of probiotic strains were clustered together on the positive axis, whereas the components in milk fermented with the 1:1, 1:5, 1:10, and 1:50 ratios were clustered together on the negative axis.

The volatile flavor compounds in the fermented milk were classified into six major types: aldehydes, ketones, acids, alcohols, esters, and aromatic hydrocarbons (Figure 1B). On the positive axis, aldehydes, ketones, alcohols, and acidic compounds were associated with the flavor of milk fermented with the 1:100 and 1:1000 ratios of probiotic bacteria. On the negative axis, esters and aromatic hydrocarbon compounds were associated with the flavor of milk fermented with the 1:1, 1:5, 1:10, and 1:50 ratios of probiotic bacteria. Aldehydes, ketones, alcohols, esters, and aromatic hydrocarbon compounds were located on the positive axis of the plane, whereas acidic compounds were located on the negative axis of the plane.

Overall, aldehydes, ketones, and alcohols were present in the samples fermented with the 1:100 and 1:1000 ratios of probiotic bacteria, indicating that a better flavor, compared with the samples fermented with the other ratios of probiotic bacteria.

### GC Fingerprint Analysis and Similarity Evaluation

**FIGURE 2:**
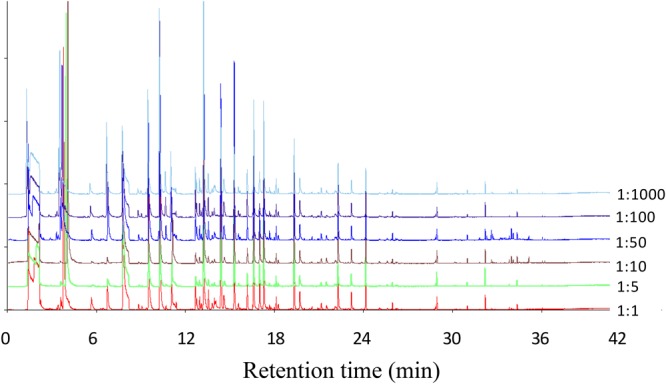
Chromatographic fingerprints of all samples of milk fermented with different ratios of *L. plantarum* P-8 to starter culture at 0 d of storage.

The GC fingerprints of six samples of milk fermented with different ratios of *L. plantarum* P-8 to starter culture were examined using the Similarity Evaluation System for Chromatographic Fingerprint of Traditional Chinese Medicines (ver. 2004A, SFDA, China) (Figure 2 and Table 2). The similarity values of all samples, prepared in triplicate, ranged from 0.923 to 0.992, indicating that all experiments had good repeatability. The similarity values between the 1:100 ratio and 1:1, 1:5, 1:10, 1:50, and 1:1000 ratio treatments were 0.59, 0.42, 0.46, 0.57, and 0.95, respectively. These values indicated higher similarity between the 1:100 and 1:1000 ratio treatments but lower similarity between the 1:100 ratio treatment and the other four ratio treatments.

**Table 2:** Similarities in the gas chromatographic fingerprints among samples treated with six different ratios (1:1, 1:5, 1:10, 1:50, 1:100, and 1:1000) of *L. plantarum* P-8 to starter culture at 0 d of storage.

	1:1	1:5	1:10	1:50	1:100	1:1000	Reference
1:1	1.00	0.61	0.76	0.68	0.59	0.55	0.85
1:5	0.61	1.00	0.61	0.62	0.42	0.42	0.76
1:10	0.76	0.61	1.00	0.79	0.46	0.43	0.84
1:50	0.68	0.62	0.79	1.00	0.57	0.55	0.86
1:100	0.59	0.42	0.46	0.57	1.00	0.95	0.80
1:1000	0.55	0.42	0.43	0.55	0.95	1.00	0.78
Reference	0.85	0.76	0.84	0.86	0.80	0.78	1.00

### Key Volatile Compounds in Fermented Milk

Generally, compounds with an OAV 0.1–1 are flavor compounds and confer an important modifying effect on the flavor of fermented milk, whereas compounds with an OAV ≥ 1 are key contributors to the flavor of fermented milk. The OAVs of volatile compounds in milk fermented with the 1:100 and 1:1000 ratios of probiotic bacteria are shown in Table 3. The odor threshold concentrations of these compounds that have been reported in the literature are presented in Table 3. The volatile compounds in the milk fermented with the 1:1, 1:5, 1:10, and 1:50 ratios consisted of 7, 7, 7, and 6 important flavor compounds, respectively. In particular, the OAV for hexanal was 5.1 in the 1:50 ratio samples, which indicated that this compound could be a significant contributor to the aroma of the fermented milk. Similar results were found in the 1:100 and 1:1000 ratio samples consisting of 10 important flavor compounds, 4 and 6 of which had OAVs of 0.1–1 and >1, respectively. Six characteristic compounds, 3-methylbutanal, hexanal, (E)-2-octenal, nonanal, 2-heptanone, and 2-nonanone, were detected in milk fermented with the 1:100 and 1:1000 ratios of probiotic bacteria. In the 1:100 and 1:1000 ratios, hexanal had an OAV of 10.99 and 10.69, respectively, which suggests that the compound could be a significant contributor to the aroma of Parmigiano-Reggiano cheese.

**Table 3:** Odor activity values (OAVs) of the compounds produced in milk fermented with 1:100 and 1:1000 ratios of *L. plantarum* P-8 to *S. thermophilus* (compared with a 1:1000 ratio of *L. delbrueckii* subsp. *bulgaricus* to *S. thermophilus*).

Volatile compound	Odor threshold (μg/L)	OAV						Reference
		1:1	1:5	1:10	1:50	1:100	1:1000	
3-Methyl-butanal	5.4	–	0.42	0.37	0.96	1.58	2.10	Qian and Reineccius, 2003
Hexanal	3	–	–	1.83	5.1	10.99	10.69	Gemert, 2003
Heptanal	750	0.002	0.0037	0.0038	0.005	0.01	0.01	Qian and Reineccius, 2003
(E)-2-Heptenal	13	–	–	0.26	–	0.79	0.71	Leffingwell and Leffingwell, 1991
(Z)-2-Heptenal	13	0.43	–	–	0.08	–	0.60	John, 2001
(E)-2-Octenal	3	1.84	1.27	1.27	1.65	3.34	2.68	John, 2001
Nonanal	1	1.03	1.05	0.63	0.56	2.38	1.99	Gemert, 2003
2-Heptanone	5	2.71	2.11	1.63	0.61	5.57	5.04	Attaie, 2009
2-Nonanone	5	2.65	1.99	1.69	0.35	4.34	3.63	Attaie, 2009
3-Methyl-1-butanol	4750	–	–	–		–	–	Qian and Reineccius, 2003
1-Octen-3-ol	10	0.13	0.11	0.08	0.05	0.23	0.26	Molimard and Spinnler, 1996
Hexanol	120	0.13	0.10	0.07	0.05	0.20	0.19	Qian and Reineccius, 2003
Acetoin	55	–	–	–	–	0.28	–	Qian and Reineccius, 2003

### pH and TA

Table 4 shows the changes in pH and TA during fermentation and storage, caused by the residual activity of microorganisms. After 2 h of fermentation, the pH of the milk began to decrease rapidly, reaching ∼4.5 in less than 6 h. In particular, the pH of the fermented milk supplemented with *L. plantarum* P-8 reached 4.01 at the end of the 14-d storage period. The TA value of the fermented milk supplemented with *L. plantarum* P-8 increased steadily during fermentation and storage, reaching 93.28°T at the end of the 14-d storage period.

**Table 4:** The physiochemical characteristics of milk fermented with a 1:100 ratio of *L. plantarum* P-8 to *S. thermophilus* (compared with a 1:1000 ratio of *L. delbrueckii* subsp. *bulgaricus* to *S. thermophilus*) during fermentation (0, 2, and 4 h) and storage (0 h, 12 h, 1 d, 2 d, 3 d, 7 d, and 14 d).

	pH		TA		Viable count (log cfu/ml)		Viscosity(mPa s)		Syneresis (%)	
Time	Lb-St-P8	Lb-St	Lb-St-P8	Lb-St	Lb-St-P8	Lb-St	Lb-St-P8	Lb-St	Lb-St-P8	Lb-St
0 h (F)	6.79 ± 0.02	6.60 ± 0.03	12.3 ± 0.15	10.83 ± 0.05	7.65 ± 0.04	7.68 ± 0.00	110 ± 1.00	112 ± 2.1	42 ± 3.1	50 ± 4.1
2 h (F)	6.24 ± 0.01	6.12 ± 0.00	18.96 ± 0.04	18.34 ± 0.13	8.26 ± 0.03	7.89 ± 0.04	110 ± 5.00	206 ± 1.8	43 ± 3.0	47 ± 3.2
4 h (F)	5.25 ± 0.00	5.6 ± 0.04	40.18 ± 0.05	40.62 ± 0.32	8.86 ± 0.00	8.34 ± 0.01	256 ± 3.00	354 ± 2.7	39 ± 0.0	41 ± 0.9
0 d (S)	4.36 ± 0.02	4.46 ± 0.01	69.7 ± 0.04	70.99 ± 0.12	9.08 ± 0.04	9.16 ± 0.03	362 ± 3.00	558 ± 2.5	35 ± 2.1	28 ± 1.2
12 h (S)	4.26 ± 0.00	4.21 ± 0.02	76.36 ± 0.08	73.79 ± 0.25	9.17 ± 0.01	9.26 ± 0.00	688 ± 4.00	986 ± 1.9	36 ± 2.1	30 ± 2.1
1 d (S)	4.23 ± 0.01	4.13 ± 0.01	79.44 ± 0.04	81.77 ± 0.31	9.57 ± 0.02	9.4 ± 0.01	720 ± 1.00	1280 ± 10.56	32 ± 3.7	29 ± 2.6
2 d (S)	4.21 ± 0.01	4.05 ± 0.00	77.9 ± 1.18	91.19 ± 0.07	9.7 ± 0.03	9.45 ± 0.03	986 ± 26.63	1146 ± 7.2	31 ± 1.1	29 ± 1.5
3 d (S)	4.24 ± 0.03	3.94 ± 0.01	79.54 ± 0.04	94.87 ± 0.16	9.72 ± 0.01	9.3 ± 0.02	1166 ± 6.00	1027 ± 9.12	31 ± 3.2	30 ± 1.0
7 d (S)	4.09 ± 0.01	3.79 ± 0.02	87.23 ± 0.23	100.34 ± 0.31	9 ± 0.04	9.19 ± 0.00	870.67 ± 4.00	834 ± 8.21	36 ± 1.3	33 ± 2.1
14 d (S)	4.01 ± 0.02	3.72 ± 0.01	93.28 ± 0.18	103.44 ± 0.17	8.25 ± 0.01	8.98 ± 0.01	870 ± 3.00	830 ± 1.05	33 ± 3.0	28 ± 1.6

*F, fermentation; S, storage.*

### Viable Cell Counts

The viable cell counts during fermentation and storage were not significantly affected by the addition of probiotics at the 1:100 ratio (Table 4). The viable cell counts in the 1:100 ratio treatment increased rapidly during fermentation (0–4 h) and storage (0–3 d), reaching 9.72 log_10_ CFU/ml after 3 d of storage, and then decreased significantly thereafter. Similar results were found in the yogurt prepared with a fixed ratio (1:1000) of *L. delbrueckii* subsp. *bulgaricus* to *S. thermophilus*, in which the viable cell counts peaked at 2 d during storage (9.45 log10 CFU/ml).

### Viscosity and Syneresis

Table 4 presents the viscosity and syneresis values of milk inoculated with the 1:100 ratio during fermentation and storage. During fermentation and storage, the viscosity of the fermented milk increased significantly over time and peaked at 1280 mPa s at 1 d of storage. Similarly, the viscosity increased steadily in the fermented milk supplemented with *L. plantarum* P-8, reaching 1166 mPa s after 3 d of storage. However, the change in viscosity during storage (at 7 and 14 d) was not significant. The fermented milk supplemented with *L. plantarum* P-8 demonstrated more syneresis than did the yogurt during refrigeration storage. Syneresis (31–36%) was observed in the fermented milk during storage.

### Sensory Assessment

The sensory evaluations of the flavor of the milk samples fermented with different *L. plantarum* P-8 to yogurt starter ratios were made by panelists at 0 d of storage. Samples fermented with *L. plantarum* P-8 to yogurt starter ratios of 1:100 were considered to have better yogurt characteristics than those of the other combinations, which were also considered to have good flavor.

## Discussion

The effect of *L. plantarum* strains as probiotic bacteria on the production of volatile aromatic compound metabolites in fermented milk has been described previously (Cheng, 2010; de Bok et al., 2011). *L. plantarum* plays an important role as a safe starter culture in food fermentation. In this study, a total of 66 volatile compounds, including aldehydes, ketones, acids, alcohols, esters, alcohols, and aromatic compounds, were identified in milk fermented with six different inoculation ratios (1:1, 1:5, 1:10, 1:50, 1:100, and 1:1000) of *L. plantarum* P-8 to *S. thermophilus* and a fixed ratio (1:1000) of *L. delbrueckii* subsp. *bulgaricus* to *S. thermophilus*.

Aldehydes have a greater impact on the flavor of fermented milk because of their lower threshold (Brányik et al., 2012). Amino acid degradation forms 3-methylbutanal, which is a potent odorant in fermented milk (Madruga et al., 2009), and 3-methylbutanal was detected in the 1:5, 1:10, 1:50, 1:100, and 1:1000 ratio treatments. High levels of 3-methylbutanal were found in milk fermented with the 1:1000 *L. plantarum* P-8 to starter culture (11.32 μg/l) ratio and 1:1000 *L. delbrueckii* subsp. *bulgaricus* to *S. thermophilus* (7.8 μg/l) treatments, indicating that 3-methylbutanal formation in fermented milk is closely related to fermentation by *L. delbrueckii* subsp. *bulgaricus* and *S. thermophilus*. Aldehydes, such as hexanal, are transitory compounds in fermented milk because they are easily reduced to acidic compounds or alcohols due to their relatively active chemical properties (Franciscojosé et al., 2010). Straight-chain aldehydes, including hexanal, heptanal, and nonanal, are quite common in fermented milk and originate from auto-oxidation of unsaturated fatty acids in milk fat. These compounds give grassy and herbaceous aromas to fermented milk. High levels of hexanal were detected in milk fermented with the 1:100 and 1:1000 ratios of bacteria (32.08 and 32.96 μg/l, respectively). Heptanal imparts a fatty aroma to fermented milk (Ferreira et al., 2000), and its maximum value (8.72 μg/l) was observed in the 1:100 ratio treatment. Heptanal levels increased with decreasing inoculation amounts of *L. plantarum* P-8, suggesting that *L. plantarum* P-8 inhibits the formation of heptanal. Nonanal has a low threshold value and provides citrus and fatty aromas to fermented milk (Piombino et al., 2008). Hexanal, heptanal, and nonanal were the most commonly observed odorants in this study and were detected in all six ratio treatments. (E)-2-Heptenal was found in milk fermented with the 1:10, 1:100, and 1:1000 ratios of probiotic bacteria, with the peak value (10.23 μg/l) at 1:100. Benzaldehyde is an important aromatic aldehyde formed from phenylacetaldehyde via α-oxidation or from cinnamic acid via β-oxidation (Dan et al., 2018). At lower levels, benzaldehyde provides an almond flavor to fermented milk, and at higher levels a fruity aroma (Chu and Yaylayan, 2008). Low levels of benzaldehyde (0.46–1.08 μg/l) were found in almost all treatment combinations, except the 1:1000 ratio. Benzaldehyde is an important compound frequently detected in dairy products such as fresh goat cheese (Condursoa et al., 2008). (E)-2-Octenal and (Z)-2-decenal were detected in milk fermented with all six ratios of bacteria, with the highest levels seen at 1:100 and 1:1000.

Ketones are produced mainly by thermal degradation of amino acids, oxidation of unsaturated fatty acids, and the Maillard reaction. As common constituents, ketones are known primarily for their effect on the aroma of most dairy products because of their low perception thresholds. A total of eight volatile ketones were detected in our milk samples. Diacetyl was detected at the beginning of fermentation (data not shown). As a byproduct of lactic acid bacteria metabolism, acetoin is produced by the chemical oxidation of diacetyl (Ott et al., 1999), which was found in milk fermented with the 1:100 ratio of probiotic bacteria. Acetoin gives fermented milk a weak creamy flavor and is an important taste compound that ameliorates the strong cream odor caused by diacetyl (Cheng, 2010). Methyl ketones including 2-heptanone, 2-nonanone, and 2-undecenone, which are known primarily for their contribution to the aroma of surface mold-ripened and blue-veined cheeses (Curioni and Bosset, 2002), were detected in our samples. As the predominant ketone compounds, 2-heptanone and 2-nonanone were detected in all six ratio treatments, with the highest levels reached at 1:100 (Pionnier and Hugelshofer, 2006; Dan et al., 2017a). 2-Undecenone was also detected in all six treatment ratios at levels ranging from 1.65 to 3.45 μg/l.

Carboxylic acids in fermented milk usually originate from lipolysis, proteolysis, or lactose fermentation (Franciscojosé et al., 2010). Studies have reported that acid compounds improve the taste of fermented milk and are the main source of sourness (Cheng, 2010). Hexanoic and heptanoic acids may be released via lipolytic activity. These short-chain fatty acids have a strong flavor; for instance, hexanoic acid gives a rancid, sweet cheese-like flavor to the fermented milk (Patton, 1964). Similar results have been reported by Chammas et al. (2006), who detected hexanoic acid in fermented milk (Chammas et al., 2006). In this study, hexanoic acid was found in the 1:1, 1:5, 1:10, and 1:50 ratio treatments, indicating that *L. plantarum* P-8 may promote the generation of hexanoic acid. Carboxylic acids are not major compounds in fermented milk due to their higher threshold values. Even though major acidic compounds were detected in all six ratio treatments, these compounds had OAV values <1 and did not significantly contribute to the overall flavor of fermented milk.

Considering the adverse effects on post-acidification and the variations in these volatile aromatics, especially acetic acid and 2-butanone as well as non-volatile metabolites, these characteristics may considerably influence the organoleptic quality of the product.

Alcohols in fermented milk may be associated with lactose metabolism, methyl ketone reduction, and amino acid metabolism (Molimard and Spinnler, 1996). High levels of 3-methylbutanol, hexanol, heptanol, and nonanol were detected in milk fermented with the different ratios of probiotic bacteria. 3-Methylbutanol can confer a pleasant aroma of fresh cheese (Galvaϸo et al., 2011), and its concentration was highest (4.43 μg/l) in milk fermented with 1:1000 ratio. Hexanol, heptanol, and nonanol are major flavor compounds in fermented milk (Cheng, 2010). These compounds were found in all six ratio treatments, with the highest levels (23.84 μg/l hexanol, 64.93 μg/l heptanol, and 9.74 μg/l nonanol) seen at the 1:100 ratio. Similar results were found in milk fermented with 1:1000 *L. delbrueckii* subsp. *bulgaricus* to *S. thermophilus* (Dan et al., 2017b). As the most common alcohol, 1-octen-3-ol has been identified as an important flavor compound in most dairy products investigated (Cheng, 2010; Ning et al., 2011); however, low levels were detected in our milk fermentation treatments. This compound has green and mushroom-like notes and contributes significantly to the aroma profiles of foods due to a low perception threshold (Curioni and Bosset, 2002).

Esters are produced primarily via the esterification of fatty acids and alcohols. Among the esters, ethyl esters have an important role in the formation of the fruity characteristics of dairy products (Curioni and Bosset, 2002). Allyl 2-ethyl butyrate as a common flavor compound was found in milk fermented with 1:1, 1:10, 1:50, and 1:100 ratios of the probiotic bacteria. Most esters provide fermented milk with fruity and floral flavors and weaken the pungent and astringent odors of fatty acids and amines (Cheng, 2010).

Aromatic hydrocarbon compounds have high flavor threshold values and do not have significant effects on the flavor of fermented milk, but at certain concentrations, they give fermented milk a fuller taste. Fifteen aromatic hydrocarbon compounds were found in all six ratio treatments and potentially play roles as supplementary flavor compounds in fermented milk.

The results of the principal component analysis and similarity evaluation revealed that the flavor of milk fermented with the 1:100 and 1:1000 ratios of probiotics was superior to the flavor of the milk prepared with the other ratios of probiotic bacteria. In this work, six key flavor compounds were found in the milk fermented with the 1:100 and 1:1000 ratios of probiotic bacteria, which were 3-methylbutanal, hexanal, (E)-2-octenal, nonanal, 2-heptanone, and 2-nonanone. All of these except for 3-methylbutanal were present in higher amounts in the treatment with a 1:100 ratio than in the treatment with a 1:1000 ratio of probiotic bacteria. In addition, acetoin was found in the milk fermented with a 1:100 ratio of probiotic bacteria. Acetoin is an important volatile compound that can influence the flavor of fermented milk. Therefore, the optimal ratio of *L. plantarum* P-8 to yogurt starter was determined to be 1:100. These results were consistent with the sensory assessment results.

The changes in the viable cell count, pH, TA, viscosity, and syneresis values in the milk fermented with the 1:100 ratio of *L. plantarum* P-8 to starter culture are shown in Table 4 during fermentation (0, 2, and 4 h) and storage (0 h, 12 h, 1 d, 2 d, 3 d, 7 d, and 14 d). The pH and TA values in fermented milk supplemented with *L. plantarum* P-8 were similar to those observed in yogurt during fermentation and storage. In this study, the pH and TA values of fermented milk supplemented with *L. plantarum* P-8 decreased or increased steadily during fermentation and storage. Similar results were obtained when milk was fermented with *S. thermophilus, L. acidophilus, Bifidobacterium* species, or *L. casei* after 35 d of refrigeration (Gilliland et al., 2010). Gueimonde et al. (2004) also reported that the pH of commercially fermented milk is between 3.9 and 4.2 (Gueimonde et al., 2004). The TA is a key indicator of the acidity of fermented milk that reflects the summed total acidic groups that include peptides and free amino-acid residues; generally, the higher the acidity, the higher the TA (Li et al., 2017). Donkor et al. (2006) reported that the taste of fermented milk improves when the TA is maintained at 70–110°T (Donkor et al., 2006). However, another study reported that consumers prefer fermented milk with a TA around 120°T (Olson and Aryana, 2008). In this study, the fermented milk pH was consistently above 4 and the acidity below 100°T during fermentation and storage, indicating that the acidity of milk fermented with our ratios of probiotic bacteria is acceptable to consumers. In general, the post-acidification of fermented milk was closely related to the lactic acid bacteria used for milk fermentation. Table 4 indicates that the milk supplemented with *L. plantarum* P-8 can delay post-acidification. These results indicated that incorporation of *L. plantarum* P-8 reduced the post-acidification of yogurt during storage.

The viable probiotic cell count is a key property of fermented milk. It is important for the milk industry to improve the number of viable bacteria in its final products. In this study, the viable cell counts in the 1:100 ratio treatment remained stable (>8.25 log CFU/g) toward the end of storage. These results are in accordance with the regulations of the International Dairy Federation, which states that the viable cell counts should exceed 10^7^ CFU/ml during the shelf life of the product. At the beginning of fermentation, the counts of *S. thermophilus* remained higher than the counts of *L. delbrueckii* subsp. *bulgaricus* (1:1000). Kneifel et al. (1993) also reported that most commercial yogurts had higher counts of *S. thermophilus* than *L. delbrueckii* subsp. *bulgaricus*. As a lactic acid-producing bacterium, *L. delbrueckii* subsp. *bulgaricus* can lead a loss in viability of *S. thermophilus* and *L. plantarum* during refrigerated storage; however, it is an essential component of the starter culture that plays critical roles in the production of lactic acid and the development of the flavor of the yogurt. Fermented milk is the most common means for the delivery of probiotic cells to the intestinal tract. The number of probiotic microorganisms in the final products is generally the most important characteristic, as probiotic products must contain an adequate amount of viable probiotic cells, which should exceed 10^6^ CFU/ml at the time of consumption (Sohrabvandi et al., 2010). In a preliminary experiment, the count of viable *L. plantarum* P-8 in the 1:100 ratio treatment was not less than 10^7^ during fermentation and storage (data not shown). He et al. (2012) also reported a similar result whereby a higher count of *L. plantarum* P-8 was detected in milk fermented with *L. plantarum* P-8 and *S. thermophilus* at various ratios.

Syneresis is the ability of fermented milk gels to bind to various components of milk, especially the water phase. Syneresis is a reversible indicator of the quality of fermented milk. Syneresis (31–36%) was observed in the fermented milk supplemented with *L. plantarum* P-8 during storage because probiotic bacteria grow slowly in basic cultures of fermented products due to the lack of proteolytic enzymes. Similar results were reported by González-Martiìnez et al. (2002), in that syneresis of yogurt supplemented with whey protein ranges from 23 to 36%. The viscosity markedly increased with fermentation time, reaching 1166 mPa s after 3 d of storage. The change in viscosity was consistent with the viable cell count in fermented milk. During fermentation and storage, the viable cell count and viscosity of the sample increased rapidly, peaking after 3 d of storage (9.72 log CFU/ml and 1166 mPa s, respectively). *L. plantarum* P-8 was reported to increase the viscosity of fermented milk, consistent with our results (Bao et al., 2012a).

## Conclusion

In this study, the quality of the fermented dairy products was determined using a starter culture and probiotics; 66 volatile flavor compounds were identified in milk fermented with six different inoculation ratios of *L. plantarum* P-8 to *S. thermophilus* and a fixed ratio (1:1000) of *L. delbrueckii* subsp. *bulgaricus* to *S. thermophilus*, including aldehydes, ketones, acids, alcohols, esters, alcohols, and aromatic compounds. There were significant changes in the volatile profiles depending on the ratio of *L. plantarum* P-8 to starter culture. Some important volatile flavor compounds, such as 3-methylbutanal, hexanal, (E)-2-octenal, nonanal, 2-heptanone, 2-nonanone, and acetoin, were identified in the 1:100 ratio treatment. In addition, the stability of milk fermented with the 1:100 ratio of *L. plantarum* P-8 to *S. thermophilus* during fermentation and storage was supported. Our results indicated that the ratio of *L. plantarum* P-8 to starter culture used is important for determination of the volatile profiles and overall flavor of the final milk products.

## Author Contributions

TD and TS designed the experiments. HC, TL, JT, and WR performed the experiments. TD, TS, and HZ drafted the manuscript. All authors read and approved the final manuscript.

## Conflict of Interest Statement

The authors declare that the research was conducted in the absence of any commercial or financial relationships that could be construed as a potential conflict of interest.
